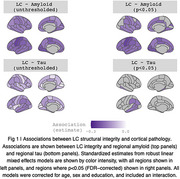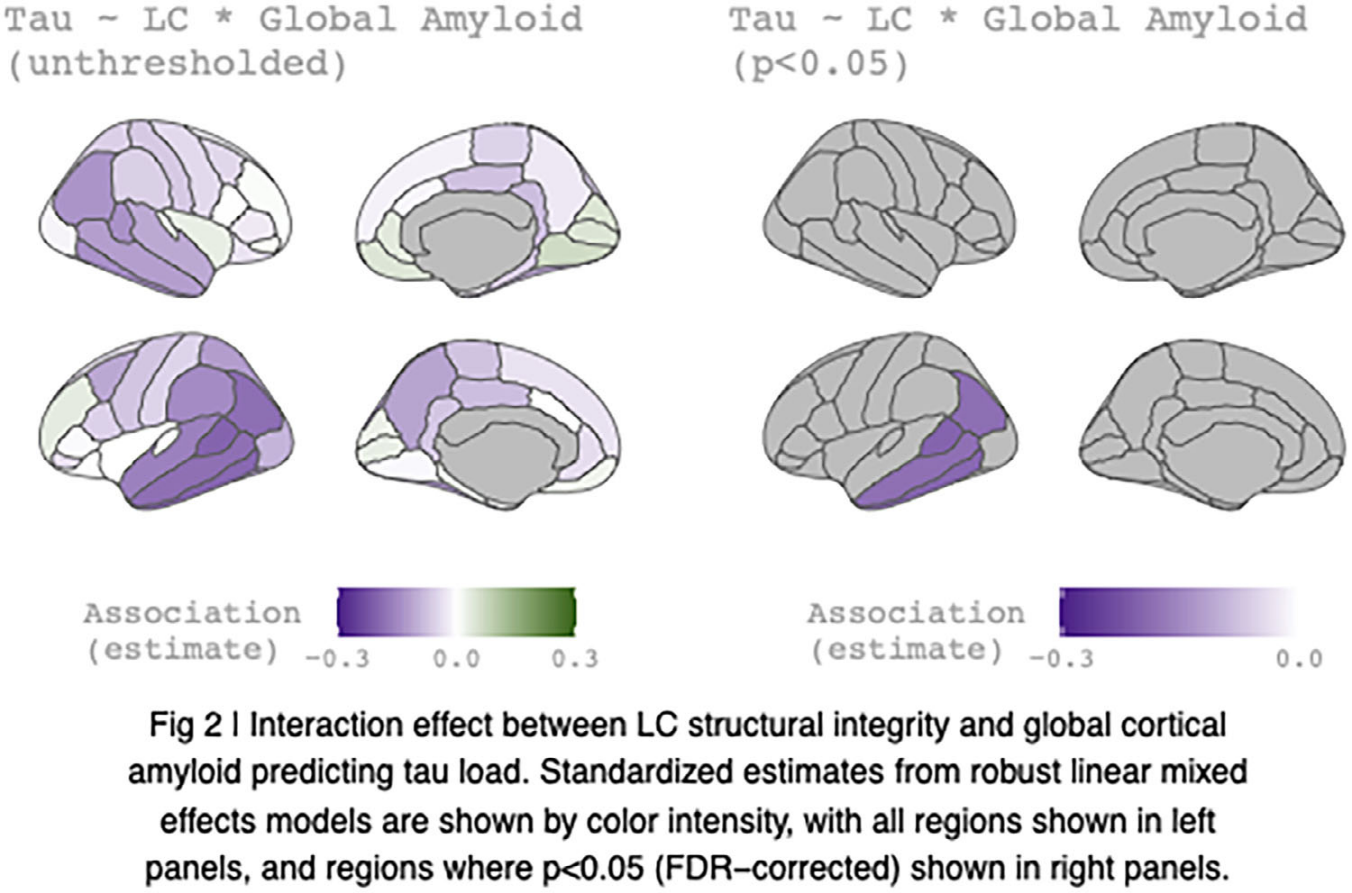# Locus coeruleus structural integrity relates to cortical amyloid and tau pathology *in vivo*

**DOI:** 10.1002/alz70855_099139

**Published:** 2025-12-23

**Authors:** Alfie Wearn, Christine L Tardif, Ilana R Leppert, Colleen S. Hughes, Giulia Baracchini, Judes Poirier, John C.S. Breitner, Gary R. Turner, Sylvia Villeneuve, R. Nathan Spreng, PREVENT‐AD Research Group

**Affiliations:** ^1^ Montreal Neurological Institute, McGill University, Montreal, QC, Canada; ^2^ Montreal Neurological Institute, Montreal, QC, Canada; ^3^ McConnell Brain Imaging Center, McGill University, Montreal, QC, Canada; ^4^ Montreal Neurological Institute‐Hospital (The Neuro), McGill University, Montreal, QC, Canada; ^5^ Brain and Mind Centre, The University of Sydney, Sydney, NSW, Australia; ^6^ Douglas Mental Health University Institute, Centre for Studies on the Prevention of Alzheimer's Disease (StoP‐AD), Montréal, QC, Canada; ^7^ York University, Toronto, ON, Canada; ^8^ McConnell Brain Imaging Centre (BIC), MNI, Faculty of Medicine, McGill University, Montreal, QC, Canada; ^9^ StoP‐AD Centre, Douglas Mental Health Institute Research Centre, Montreal, QC, Canada; ^10^ Douglas Mental Health University Institute, Montreal, QC, Canada

## Abstract

**Background:**

The locus coeruleus (LC) is one of the first epicentres of tau pathology in Alzheimer's disease, displaying pathology decades before clinical symptom onset. We investigated whether LC integrity was associated with cortical deposition of amyloid and tau *in vivo* in asymptomatic, at‐risk older adults.

**Method:**

208 healthy older adults with first‐degree familial history of Alzheimer's disease were included from the PREVENT‐AD cohort (mean age 68.4 ± 5.14y, 69% female). 155 subjects were also examined longitudinally over 38.8 ± 14.0 months.

Subjects underwent longitudinal 3T MRI: 1mm^3^ T1w MPRAGE and Neuromelanin‐sensitive brainstem imaging (0.7x0.7x1.8mm). Cortical amyloid and tau were quantified using PET imaging (single timepoint), with 18F‐NAV4694 and 18F‐flortaucipir tracers, respectively. Regional specific uptake value ratios (SUVR) were calculated for each, relative to cerebellar grey matter.

LC was automatically delineated on individual neuromelanin‐sensitive brainstem scans by identifying 10 brightest connected voxels within an approximate region of interest and calculating contrast‐to‐noise relative to a pontine control region (LC_CNR_).

We tested associations between LC_CNR_ and regional amyloid and tau SUVR using robust linear mixed effects models for each region with random intercepts across subjects. Longitudinal change was tested as an interaction term between the PET measure and time. We also tested the dependence of the Tau‐LC association on global amyloid pathology at baseline using linear regression. All analyses were corrected for baseline age, sex and education and *p*‐values were FDR‐adjusted.

**Results:**

We observed negative associations between LC integrity and amyloid SUVR across almost all cortical regions (Figure 1). Associations between LC integrity and cortical tau were restricted to temporal lobes, including parahippocampal gyrus, and inferior parietal regions (Figure 1). Associations with both amyloid and tau were particularly strong in the left hemisphere. These associations were observed only at baseline, with no longitudinal effects observed.

The relationship between baseline LC integrity and cortical tau was modulated by global amyloid in lateral temporal cortices, particularly along in the left lateral temporal cortex (Figure 2).

**Conclusion:**

LC degeneration was associated with greater expression of key hallmarks of AD pathology in asymptomatic older adults. We support the model that LC is central to the earliest pathophysiological changes of AD.